# Association between exposure to air pollution during pregnancy and false positives in fetal heart rate monitoring

**DOI:** 10.1038/s41598-017-12663-2

**Published:** 2017-09-29

**Authors:** Seiichi Morokuma, Takehiro Michikawa, Shin Yamazaki, Hiroshi Nitta, Kiyoko Kato

**Affiliations:** 1 Department of Obstetrics and Gynaecology, Kyushu University Hospital, Kyushu University, 3-1-1 Maidashi, Higashi-ku, Fukuoka 812-8582 Japan; 2 Environmental Epidemiology Section, Centre for Health and Environmental Risk Research, National Institute for Environmental Studies, 16-2 Onogawa, Tsukuba, Ibaraki 305-8506 Japan

## Abstract

Fetal heart rate (FHR) monitoring is essential for fetal management during pregnancy and delivery but results in many false-positive diagnoses. Air pollution affects the uterine environment; thus, air pollution may change FHR reactivity. This study assessed the association between exposure to air pollution during pregnancy and FHR monitoring abnormalities using 2005–2010 data from the Japan Perinatal Registry Network database. Participants were 23,782 singleton pregnant women with FHR monitoring, without acidemia or fetal asphyxia. We assessed exposure to air pollutants, including particulate matter (PM), ozone, nitrogen dioxide (NO_2_), and sulfur dioxide (SO_2_). In a multi-trimester model, first-trimester PM exposure was associated with false positives in FHR monitoring (odds ratio [OR] per interquartile range (10.7 μg/m^3^) increase = 1.20; 95% CI: 1.05–1.37), but not second-trimester exposure (OR = 1.05; 95% CI: 0.91–1.21) and third-trimester exposure (OR = 1.06; 95% CI: 0.96-1.17). The association with first-trimester PM exposure persisted after adjustment for exposure to ozone, NO_2_, and SO_2_; however, ozone, NO_2_, and SO_2_ exposure was not associated with false positives in FHR monitoring. First-trimester PM exposure may alter fetal cardiac response and lead to false positives in FHR monitoring.

## Introduction

Research on fetal heart rate (FHR) monitoring has been conducted to determine the clinical significance of different morphological categories of FHR tracing. It was thought that if hypoxia and other aspects of fetal status could be understood and medical interventions administered early, the incidence of fetal death during delivery, neonatal asphyxia, and even cerebral palsy could be reduced^1^. However, subsequent studies reported no change in the incidence of cerebral palsy, and while the number of perinatal deaths has declined, the number of cesarean sections (C-sections) has increased^2^. Abnormalities in FHR monitoring can appear as late deceleration (LD), severe variable deceleration (SVD), loss of variability (LV), or bradycardia. When these occur alone or in combination, non-reassuring fetal status is diagnosed, and immediate delivery of the fetus is recommended^3^. However, the increase in the C-section rate has been accompanied by an increased awareness of the harm this procedure can cause. Moreover, fetuses that exhibit FHR abnormalities do not necessarily present with acidosis and do not always require immediate delivery^3–5^. In other words, there are many cases of false positives in FHR monitoring which do not actually present with acidosis. Since FHR monitoring is an essential component of fetal management, pursuing the causes of false positives in FHR monitoring is extremely important. However, these causes are not clearly understood^6–8^.

Recently, air pollution has been linked to fetal growth restriction, low birth weight^9–11^, and preterm birth^12^. Research in animal models has confirmed that air pollution can damage the uterine environment and affect fetal growth and development^13,14^. Fetuses adapt to changes in the uterine environment (e.g., undernutrition, maternal inflammation, and stress), and the results of these adaptations may manifest in adulthood as heart disease or other health problems^15^. Air pollution can cause uterine inflammation^16^, which often leads to hypoxic states^17^. In animal experiments, hypoxic stimulation causes changes in heart rate responses. Subsequently, the same amount of hypoxic stimulation caused greater declines in the heart rate^18,19^.

Therefore, we hypothesized that exposure to inflammation caused by air pollution changes the fetal heart rate responses and causes false positives in FHR monitoring. In this study, we considered cases that presented with FHR monitoring abnormalities, without acidemia or fetal asphyxia, with umbilical arterial blood pH ≥ 7.2, and Apgar score ≥7, as false positives^20,21^, and examined the association with exposure to air pollution during pregnancy.

## Methods

### Study population

This study was approved by the Institutional Review Board of Kyushu University (No. 28-153), Japan, and the Japan National Institute for Environmental Studies (No. 1-2016-005) and performed according to the Declaration of Helsinki. Analyses were performed on de-identified data from the Japan Perinatal Registry Network database, which is maintained by the Japan Society of Obstetrics and Gynecology. This database includes data on all live births and stillbirths after 22 weeks of gestation at participating hospitals (primarily university hospitals and local general hospitals). Details of this database have been published previously^22^.

In this study, we used perinatal data from 28 hospitals across eight prefectures (Fukuoka, Saga, Nagasaki, Kumamoto, Oita, Miyazaki, Kagoshima, and Okinawa) in the western part of Japan (Kyushu-Okinawa District), from 2005 to 2010. The data included maternal age, height, weight, parity, gestational age as determined from ultrasound findings in early pregnancy, smoking status and alcohol consumption during pregnancy, medical history, diagnoses of obstetric complications, birth weight, and infant sex.

During the study period, 47,835 singleton deliveries were registered. Among these, 6,906 deliveries for which the prefecture of the mother’s residence was different from the location of the birth hospital were excluded to avoid exposure misclassification due to the Japanese satogaeri custom (some mothers go back to their parental homes, and deliver at a hospital near their parents’ homes). In addition, the following deliveries were excluded: 2,832 deliveries with gestational age <32 weeks (unsuitable for FHR assessment^23,24^); 9,752 deliveries with missing information on umbilical cord blood pH (n = 7,282) or umbilical blood pH < 7.2 (acidemia)^20,25^ (n = 2,470); 1,385 deliveries with Apgar scores <7 at 1 minute or 5 minutes after birth (to restrict the study sample to newborns in good health^21^); 1,726 deliveries to mothers with intrauterine infection, oligohydramnios, or placental abruption (which are causes of FHR monitoring abnormalities^26,27^; one delivery with fetal death (stillbirth); 1,346 deliveries with conception date ≥22 weeks before the study period began and those with conception dates ≤42 weeks before the study period ended to prevent fixed cohort bias^28^, and 105 deliveries with missing data on maternal age (n = 2) or exposure to pollutants (n = 103). After applying these exclusions, 23,782 singleton deliveries at gestational age ≥32 weeks were included in the analysis.

### Environmental data

Concentrations of air pollutants (particulate matter [PM], nitrogen dioxide [NO_2_], sulfur dioxide [SO_2_], and ozone measured as photochemical oxidants), measured at ambient air pollution monitoring stations and whose measurements were regarded to be representative of their respective communities, were collected from the Japan National Institute for Environmental Studies’ atmospheric environment database. As an indicator of PM, we used suspended PM, defined under the Japan Air Quality Standards as airborne particles with a 100% cut-off level of 10-μm aerodynamic diameter^29^. Because routine measurement of PM_2.5_ (particles with a 50% cut-off level of 2.5 μm in aerodynamic diameter) at monitoring stations did not start in Japan until after 2010, we could not assign PM_2.5_ concentrations to most of the study participants.

The Japan Perinatal Registry Network database includes de-identified data; thus, we did not have the specific residential addresses or postal codes of study participants. Based on the assumption that the pregnant women reside near their delivery hospitals, which we previously validated^30^, we assigned pollutant concentrations measured at the closest monitoring station to each participant’s delivery hospital. Each monitoring station and the corresponding hospital were located within the same administrative area, and the median linear distance between them was 1.1 miles (1.8 km), except for one hospital in Okinawa prefecture (8.5 miles [13.6 km]). Specific locations of 24 monitoring stations and 28 hospitals have been described previously^31^.

We used daily mean concentrations of air pollutants (maximum 8-h mean concentrations for ozone) and calculated the average concentrations during the first trimester (0–13 weeks of gestation), second trimester (14–27 weeks of gestation), and third trimester (28-31 weeks of gestation as we included women who delivered in the preterm period).

### FHR monitoring abnormality

FHR monitoring was performed at the time of hospitalization after the onset of labor or for complications such as the rupture of membranes. FHR monitoring was also performed during delivery.

The Japan Perinatal Registry Network database contains data on the following eight intrapartum FHR monitoring parameters: early deceleration (ED), LD, mild variable deceleration (MVD), SVD, LV, bradycardia, tachycardia, and others. In the present study, FHR monitoring abnormalities was defined as LD, SVD, LV, or bradycardia alone or a combination of them; ED is considered to be a normal reaction, and MVD and tachycardia are not attributable to acidosis alone^32^. However, if ED, MVD, tachycardia or others were recorded in addition to LD, SVD, LV, or bradycardia, the participant was still classified under the FHR monitoring abnormality category. Concerns could also be raised regarding the reliability and validity of diagnoses based on FHR monitoring. However, as assessments were performed by obstetricians at university hospitals and regional general hospitals, we expected inter-rater variability to be low^33^.

### Statistical analysis

To measure the association between exposure to pollutants during the first trimester and FHR monitoring abnormalities, odds ratios (ORs) and 95% confidence intervals (CIs) per 10-unit increase in pollutant concentrations were estimated using multilevel logistic regression with hospital-level random effects (Supplementary figure). In this study, we assumed a linear association, since there was no evidence of a non-linear one. Regression models were adjusted for exposure to each pollutant during the first, second and third trimesters; maternal age at delivery (<25, 25–29, 30–34, ≥35 years); birth year (2005, 2006, 2007, 2008, 2009, 2010); season of conception (spring, summer, autumn, winter); smoking during pregnancy (no, yes, missing); alcohol drinking during pregnancy (no, yes, missing); parity (0, ≥1); mode of delivery (vaginal, scheduled cesarean, emergency cesarean); gestational age at birth (^32–36^, ≥37 weeks of gestation); small for gestational age (birth weight <10 percentile for gestational age) (yes, no) due to abnormal FHR based on prematurity^23,24^; and premature rupture of membranes (yes, no) and presence of congenital abnormalities (yes, no) as these two conditions are associated with FHR monitoring abnormalities^34–37^. Then, we constructed a multi-trimester model including exposure during all three trimesters. In addition, we performed several sensitivity analyses in order to avoid the possibility of residual confounding. We also constructed a multi-pollutant model including exposure to all pollutants. Statistical analyses were conducted using STATA 13 for Windows (Stata Corporation, College Station, TX, USA).

## Results

The characteristics of the 23,782 deliveries included in the study are shown in Table 1. The mean maternal age at delivery was 31.0 years (standard deviation [SD] = 5.5), and 9.8% of fetuses exhibited FHR monitoring abnormalities. During the first trimester, mean concentrations of the pollutants were 27.1 μg/m^3^ (SD =8.0) for PM, 41.3 ppb (9.7) for ozone, 11.5 ppb (6.2) for NO_2_, and 3.1 ppb (1.4) for SO_2_ (Table 2). Pearson’s correlation coefficients for exposure to PM and NO_2_ were moderate (0.52 during the first trimester, 0.54 during the second trimester, and 0.47 during the third trimester).

**Table 1:** Characteristics of study participants from western Japan, 2005–2010 (n = 23,782).

Variables	n^a^	%
Maternal age at delivery (years)		
<25	3,102	13.0
25–29	6,001	25.2
30–34	8,011	33.7
≥35	6,668	28.0
Birth year		
2005	2,111	8.9
2006	3,663	15.4
2007	3,756	15.8
2008	3,801	16.0
2009	5,104	21.5
2010	5,347	22.5
Season of conception		
Spring (Mar–May)	6,599	27.8
Summer (Jun–Aug)	5,347	22.5
Autumn (Sep–Nov)	5,558	23.4
Winter (Dec–Feb)	6,278	26.4
Parity		
0	10,550	44.4
≥1	13,229	55.6
Smoking during pregnancy		
No	15,349	90.1
Yes	1,689	9.9
Alcohol drinking during pregnancy		
No	15,805	94.5
Yes	939	5.6
Gestational age at birth (weeks)		
32–36	3,374	14.2
≥37	20,408	85.8
Premature rupture of membranes		
Yes	2,285	9.6
No	21,497	90.4
Mode of delivery		
Vaginal	15,091	63.5
Scheduled cesarean	5,076	21.3
Emergency cesarean	3,615	15.2
Presence of congenital anomalies		
Yes	787	3.3
No	22,995	96.7
Small for gestational age (birth weight <10 percentile for gestational age)		
Yes	2,179	9.2
No	21,570	90.8
Fetal heart rate monitoring abnormalities		
Yes	2,323	9.8
No	21,459	90.2

^a^Numbers in the subgroups do not tally with the overall number because of missing data.

**Table 2:** Summary of average concentrations of each air pollutant and Pearson's correlations between pollutants in western Japan, 2005–2010.

Pollutant	Pollutant	n	Mean (SD^a^)	Percentile				Pearson's correlation			
				25	50	75	Interquartile range	PM^b^ (μg/m^3^)*	Ozone (ppb)	NO_2_^c^ (ppb)	SO_2_^d^ (ppb)
Concentrations during the first trimester (0–13 weeks of gestation)											
	PM (μg/m^3^)^e^	23,782	27.1 (8.0)	21.4	27.2	32.1	10.7	1			
	Ozone (ppb)^f^	21,655	41.3 (9.7)	34.5	40.2	47.9	13.4	0.12	1		
	NO_2_ (ppb)	21,543	11.5 (6.2)	7.0	11.5	16.2	9.2	0.52	−0.17	1	
	SO_2_ (ppb)	23,477	3.1 (1.4)	2.0	2.9	3.9	1.9	0.17	−0.16	0.37	1
Concentrations during the second trimester (14–27 weeks of gestation)											
	PM (μg/m^3^)	23,782	26.9 (7.8)	21.4	27.1	31.8	10.4	1			
	Ozone (ppb)	21,655	42.0 (9.4)	35.1	41.0	48.3	13.2	0.11	1		
	NO_2_ (ppb)	21,543	11.2 (6.0)	6.7	11.2	15.8	9.1	0.54	−0.16	1	
	SO_2_ (ppb)	23,477	3.0 (1.4)	2.0	2.8	3.9	1.9	0.16	−0.16	0.35	1
Concentrations during the second trimester (28–31 weeks of gestation)											
	PM (μg/m^3^)	23,623	27.0 (9.2)	20.4	26.4	32.8	12.4	1			
	Ozone (ppb)	21,628	41.6 (12.7)	32.4	40.2	50.4	18.0	0.18	1		
	NO_2_ (ppb)	21,346	11.0 (6.2)	6.2	10.9	15.4	9.2	0.47	-0.09	1	
	SO_2_ (ppb)	23,357	2.9 (1.6)	1.8	2.7	3.9	2.1	0.21	-0.03	0.32	1

^a^SD = standard deviation, ^b^PM = particulate matter, ^c^NO_2_ = nitrogen dioxide, ^d^SO_2_ = sulfur dioxide,

^e^As an indicator of PM, we used suspended PM, defined as airborne particles with a 100% cut-off level of 10-μm aerodynamic diameter under the Japan Air Quality Standards.

^f^ppb = parts per billion.

Associations between exposure to pollutants and false positives in FHR monitoring are shown in Table 3. In a multi-trimester model, first-trimester PM exposure was positively associated with false positives in FHR monitoring (OR per interquartile range (10.7 μg/m^3^) increase in PM concentrations = 1.20; 95% CI: 1.05–1.37). This association was not significantly changed after exposure to ozone, NO_2_, or SO_2_ during the first trimester (OR = 1.23, 95% CI = 1.05–1.44) (Table 4). In contrast, no significant association was observed between exposure in other trimesters and false positives in FHR monitoring (OR per interquartile range (10.4 μg/m^3^) = 1.05, 95% CI: 0.91–1.21 for the second trimester; and OR per interquartile range (12.4 μg/m^3^) = 1.06, 95% CI: 0.96-1.17 for the third trimester). The results for first-trimester PM exposure were robust in all the sensitivity analyses as follows: excluding participants in the Okinawa area due to the distance between one hospital and its respective monitoring station; restricted to nulliparous women; restricted to non-smokers and non-drinkers; excluding women who delivered in the preterm period (^32–36^ weeks of gestation) or who delivered a small for gestational age infant; excluding women with premature rupture of membranes or those who delivered an infant with congenital anomalies; and excluding women with scheduled cesarean deliveries to exclude births with less delivery-related stress to the fetus. Exposure to ozone, NO_2_, or SO_2_ during the first, second, and third trimesters were not associated with false positives in FHR monitoring.

**Table 3:** Odds ratios (ORs) and 95% confidence intervals (CIs) for the association between exposure to pollutants and fetal heart rate monitoring abnormalities in western Japan, 2005–2010.

	n	No. of outcome	OR per interquartile range increase	95% CI	
Exposure during the first trimester					
PM ^a,b^					
Model 1^c^	23,782	2,323	1.22	1.07	1.38
Model 2^d^	23,623	2,303	1.20	1.05	1.37
Ozone					
Model 1	23,655	1,975	1.02	0.93	1.11
Model 2	21,628	1,972	1.01	0.92	1.11
NO_2_^e^					
Model 1	21,543	2,014	0.83	0.66	1.04
Model 2	21,346	2,000	0.79	0.62	1.01
SO_2_^f^					
Model 1	23,477	2,272	1.02	0.91	1.13
Model 2	23,357	2,260	0.98	0.87	1.11
Exposure during the second trimester					
PM					
Model 1	23,782	2,323	1.12	0.99	1.27
Model 2	23,623	2,303	1.05	0.91	1.21
Ozone					
Model 1	23,655	1,975	0.97	0.89	1.07
Model 2	21,628	1,972	0.99	0.90	1.09
NO_2_					
Model 1	21,543	2,014	0.90	0.72	1.13
Model 2	21,346	2,000	1.08	0.83	1.41
SO_2_					
Model 1	23,477	2,272	1.06	0.95	1.18
Model 2	23,357	2,260	1.12	0.97	1.29
Exposure during the third trimester					
PM					
Model 1	23,623	2,303	1.07	0.97	1.17
Model 2	23,623	2,303	1.06	0.96	1.17
Ozone					
Model 1	21,628	1,972	1.07	0.98	1.18
Model 2	21,628	1,972	1.06	0.97	1.17
NO_2_					
Model 1	21,346	2,000	0.86	0.71	1.04
Model 2	21,346	2,000	0.82	0.65	1.02
SO_2_					
Model 1	23,357	2,260	0.98	0.89	1.08
Model 2	23,357	2,260	0.93	0.83	1.05

^a^PM = particulate matter.

^b^As an indicator of PM, we used suspended PM, defined as airborne particles with a 100% cut-off level of 10-μm aerodynamic diameter under the Japan Air Quality Standards.

^c^Adjusted for maternal age at delivery, birth year, season of conception, parity, smoking during pregnancy, alcohol drinking during pregnancy, gestational age at birth, premature rupture of membranes, mode of delivery, presence of congenital anomalies, and small for gestational age.

^d^We included exposure to pollutant during the first, second, and third trimesters simultaneously.

^e^NO_2_ = nitrogen dioxide, ^f^ SO_2_ = sulfur dioxide.

**Table 4:** Sensitivity analyses of the association between exposure to particulate matter (PM)^a^ during the first trimester and fetal heart rate monitoring abnormalities

	n	No. of outcome	OR^b^ per interquartile range increase^c^	95% CI^d^	
Model 2 in Table 3	23,623	2,323	1.20	1.05	1.37
Adjusted for exposure to Ozone, NO_2_, and SO_2_ during the first trimester	19,215	1,623	1.23	1.05	1.44
Excluding Okinawa area	20,263	1,997	1.26	1.06	1.49
Restricted to nulliparous	10,503	1,483	1.25	1.03	1.52
Restricted to non-smokers during pregnancy	15,212	1,507	1.29	1.08	1.54
Restricted to non-alcohol drinkers during pregnancy	15,665	1,549	1.21	1.02	1.44
Excluding women who delivered preterm (32–36 weeks of gestation)	20,276	1,874	1.22	1.03	1.45
Excluding women who delivered a baby small for gestational age	21,419	1,952	1.28	1.08	1.51
Excluding women with premature rupture of membranes	21,352	2,037	1.24	1.05	1.47
Excluding women who delivered a baby with congenital anomalies	22,838	2,216	1.26	1.08	1.48
Excluding women with scheduled cesarean deliveries	18,575	2,274	1.23	1.05	1.44

^a^As an indicator of PM, we used suspended PM, defined as airborne particles with a 100% cut-off level of 10-μm aerodynamic diameter under the Japan Air Quality Standards.

^b^OR = odds ratio.

^c^Adjusted for maternal age at delivery, birth year, season of conception, parity, smoking during pregnancy, alcohol drinking during pregnancy, gestational age at birth, premature rupture of membranes, mode of delivery, presence of congenital anomalies, small for gestational age, and exposure to PM during the second and third trimesters.

^d^CI = confidence interval.

## Discussion

In this study, we observed a positive association between exposure to PM in the first trimester of pregnancy and false positives in FHR monitoring. No associations were observed for ozone, NO_2_, or SO_2_. Moreover, no association was observed between PM exposure in the second- and third-trimester and false positives in FHR monitoring. To the best of our knowledge, this is the first study to report an association between first-trimester PM exposure and false positives in FHR monitoring.

False positives in FHR monitoring have been linked to congenital anomalies, infections, and maternal smoking^35–39^. FHR monitoring abnormalities have been reported at high frequencies in these cases, even when the fetus was otherwise well. Although FHR monitoring abnormalities can lead to emergency C-sections in cases of cardiac malformation, acidosis is often not observed in such cases. Moreover, bradycardia, LD, and other FHR monitoring abnormalities are frequently observed in cases of cytomegalovirus infection^38^. Additionally, maternal smoking can alter the regulation of heart rate and increase infant reactivity soon after birth^39,40^. Even in the absence of complications, maternal smoking has been linked to an elevated C-section rate due to the diagnosis of non-reassuring fetal status^39^. While the study reporting this association did not measure umbilical arterial blood pH, no differences in Apgar scores were reported between the smoking and non-smoking groups^39^. Although exposure to maternal smoking can occur throughout gestation, infections have a greater influence on the fetus early in pregnancy. FHR changes are reciprocally formed and regulated by the autonomic nervous system, the cardiovascular center in the brainstem, and the cardiac conduction system^41^. The first trimester is a critical period for the formation of these control mechanisms. In the present study, the association between false positives in FHR monitoring and exposure to air pollution was observed for the first-trimester but not second or third-trimester exposure. Nachman *et al*.^16^ reported a direct association between PM_2.5_ exposure and intrauterine inflammation using histopathological analyses of human placenta samples. On the one hand, inflammatory tissue often leads to hypoxic states^17^. In animal experiments, hypoxic stimulation causes changes in heart rate responses. Subsequently, the same amount of hypoxic stimulation caused greater declines in the heart rate^18,19^. Based on these findings, about the potential mechanism, we thought that uterine inflammation and hypoxic stimulation caused by air pollution in the first trimester might affect the cardiovascular center of the brainstem and the heart rate reactivity of the fetus. However, further research regarding the underlying mechanism is needed.

One limitation of this study is the locations at which air pollution levels were measured. Data were collected from the ambient air pollution monitoring station closest to the hospital where the study participant gave birth and thus provided imperfect measurements of women’s individual exposures. However, this exposure misclassification was likely to be non-differential; therefore, we would underestimate the effect of PM exposure on abnormal FHR. And there are also a limitation of this study. Because routine measurement of PM2.5 at monitoring stations did not start in Japan until after 2010, we could not analyze PM 2.5 separately in this study. In addition, since there were unmeasured factors, such as socioeconomic status, the possibility of residual confounding could not be excluded. Finally, the generalization of our results requires careful attention because of the exclusion of women thought to have participated in the *satogaeri* custom.

In 33.5% (777) of the cases that showed false positives in FHR monitoring, emergency C-sections were performed. Hence, preventing air pollution exposure in the first trimester of pregnancy could help reduce C-sections performed because of false positives in FHR monitoring. This is highly significant as it could help conserve medical resources. We recommend that future epidemiological studies be performed to verify and build on these findings.

## Conclusions

We observed a positive association between exposure to PM in the first trimester and false positives in FHR monitoring. No associations were observed for ozone, NO_2_, or SO_2_. Moreover, no association was observed between PM exposure in the second- and third-trimester and false positives in FHR monitoring. Thus, first-trimester PM exposure may alter the FHR response and lead to false positives in FHR monitoring.

## Electronic supplementary material

Supplementary figure
