# Supplementary material for: Association between exposure to air pollution during pregnancy and false positives in fetal heart rate monitoring
Source: Sci Rep. 2017 Sep 29;7:12421. doi: 10.1038/s41598-017-12663-2 (PMC5622039; doi:10.1038/s41598-017-12663-2)
Supplement: Supplementary file 1 — Supplementary figure [file 41598_2017_12663_MOESM1_ESM.pdf]

Title:

Association between exposure to air pollution during pregnancy and false positives in fetal heart rate monitoring

Authors:

Seiichi Morokuma[a], Takehiro Michikawa[b], Shin Yamazaki[b], Hiroshi Nitta[b], Kiyoko Kato[a]

[a]Department of Obstetrics and Gynaecology, Kyushu University Hospital, Kyushu University, 3-1-1 Maidashi, Higashi-ku, Fukuoka 812-8582, Japan

[b]Environmental Epidemiology Section, Centre for Health and Environmental Risk Research, National Institute for Environmental Studies, 16-2 Onogawa, Tsukuba, Ibaraki 305-8506, Japan

Supplementary figure.

The equation for multilevel logistic regression.

$$\log\left(\frac{p_{ij}}{1-p_{ij}}\right) = \beta_0 + \sum_{k=1}^{r}\left(\beta_k X_{ijk}\right) + u_i$$

$\frac{p_{ij}}{1-p_{ij}}$ is the odds of abnormal fetal heart rate for participant j in hospital i

β_0 is the intercept.

β_k is regression coefficients corresponding to each co-variate.

X_{ijk} is an individual value of co-variates.

u_i is a hospital-level random effect.
